# Supplementary figures and images for: Three years of insecticide resistance evolution and associated mechanisms in Aedes aegypti populations of Ouagadougou, Burkina Faso
Source: PLoS Negl Trop Dis. 2024 Dec 2;18(12):e0012138. doi: 10.1371/journal.pntd.0012138 (PMC11637278; doi:10.1371/journal.pntd.0012138)

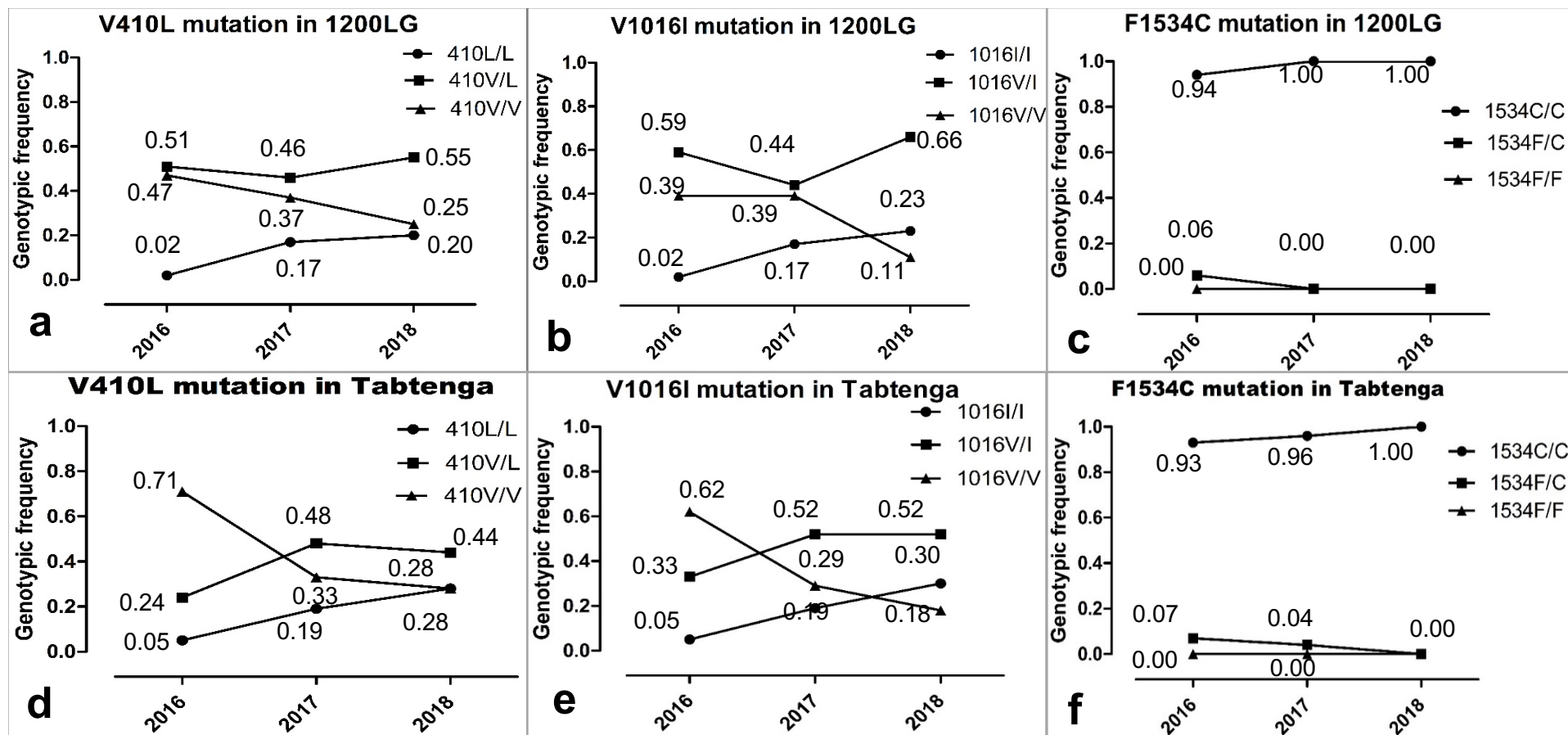

Supplement: S1 Fig — (PDF) [file pntd.0012138.s001.pdf]
