# Supplementary material for: Three years of insecticide resistance evolution and associated mechanisms in Aedes aegypti populations of Ouagadougou, Burkina Faso
Source: PLoS Negl Trop Dis. 2024 Dec 2;18(12):e0012138. doi: 10.1371/journal.pntd.0012138 (PMC11637278; doi:10.1371/journal.pntd.0012138)
Supplement: S2 Table — The numbers of each genotype are provided, along with frequencies in parentheses. The probability for comparison of the genotype distributions over the three years for each locality was determined using the Fisher Exact test. (DOCX) [file pntd.0012138.s003.docx]

**S1 Table**: Co-occurrence of F1534C, V1016I and V410L mutations in 286 samples of *Ae. aegypti* populations*.* The numbers of each genotype are provided, along with frequencies in parentheses. The probability for comparison of the genotype distributions over the three years for each locality was determined using the Fisher Exact test.

| Genotypes | 1200LG | | | Tabtenga | | |
| --- | --- | --- | --- | --- | --- | --- |
|  | 2016 | 2017 | 2018 | 2016 | 2017 | 2018 |
| CIL/CIL | 1 (0.020) | 8 (0.22) | 9 (0.20) | 2 (0.04) | 10 (0.19) | 12 (0.24) |
| CIV/CIL | 0 (0.00) | 0 (0.00) | 1 (0.02) | 0 (0.00) | 1 (0.02) | 2(0.08) |
| CIV/CIV | 0 (0.00) | 0 (0.00) | 0 (0.00) | 0 (0.00) | 1 (0.02) | 1 (0.08) |
| CVL/CIL | 0 (0.00) | 0 (0.00) | 0 (0.00) | 0 (0.00) | 0 (0.00) | 2 (0.04) |
| CVV/CIL | 23 (0.47) | 10 (0.28) | 23 (0.52) | 10 (0.23) | 23 (0.44) | 20 (0.40) |
| CVV/CIV | 4 (0.08) | 0 (0.0) | 6 (0.14) | 4 (0.09) | 1 (0.02) | 1 (0.02) |
| CVV/CVL | 0 (0.00) | 1 (0.03) | 0 (0.00) | 0 (0.00) | 0 (0.00) | 0 (0.00) |
| CVV/CVV | 18 (0.37) | 17 (0.47) | 5 (0.11) | 26 (0.58) | 14 (0.27) | 9 (0.18) |
| FVV/CIL | 2 (0.04) | 0 (0.00) | 0 (0.00) | 1 (0.02) | 1 (0.02) | 0 (0.00) |
| FVV/CVV | 1 (0.02) | 0 (0.00) | 0 (0.00) | 2 (0.04) | 1 (0.02) | 0 (0.00) |
| Fisher exact test | P<<0.001 | | | P=0.001 | | |
