# Supplementary material for: Three years of insecticide resistance evolution and associated mechanisms in Aedes aegypti populations of Ouagadougou, Burkina Faso
Source: PLoS Negl Trop Dis. 2024 Dec 2;18(12):e0012138. doi: 10.1371/journal.pntd.0012138 (PMC11637278; doi:10.1371/journal.pntd.0012138)
Supplement: S3 Table — For each predictor, the incidence rate ratio, confidence limits and corresponding p-values are provided. (DOCX) [file pntd.0012138.s004.docx]

**S2 Table**: Glm of *survivor* genotypes in *Ae. aegypti* to permethrin and deltamethrin from 1200LG and Tabtenga collected in 2017 and 2018. For each predictor, the incidence rate ratio, confidence limits and corresponding p-values are provided.

| Predictors | Odds Ratios | CI | P |
| --- | --- | --- | --- |
| (Intercept) | 0.45 | 0.15 – 1.25 | 0.138 |
| Insecticide [delta] |  |  |  |
| perm | 17.20 | 3.66 – 133.92 | 0.001 |
| Locality [1200LG] |  |  |  |
| Tab | 0.50 | 0.14 – 1.65 | 0.259 |
| Year [2017] |  |  |  |
| 2018 | 39.11 | 5.19 – 943.55 | 0.003 |
| Genotypes [VV/VV] |  |  |  |
| IL/IL | 6.47 | 1.62 – 30.66 | 0.012 |
| IV/IL | – | – | 0.996 |
| IV/IV |  | – | – |
| VL/IL | – | – | 0.998 |
| VV/IL | 1.50 | 0.50 – 4.55 | 0.467 |
| VV/IV | 0.21 | 0.02 – 2.43 | 0.191 |
| VV/VL | – | – | – |
| Insecticide [delta] × Locality [1200LG] |  |  |  |
| perm × Tab | 0.31 | 0.03 – 2.32 | 0.275 |
| Locality [1200LG] × Year [2017] |  |  |  |
| Tab × 2018 | 2.18 | 0.07 – 69.76 | 0.622 |
