# Supplementary material for: Three years of insecticide resistance evolution and associated mechanisms in Aedes aegypti populations of Ouagadougou, Burkina Faso
Source: PLoS Negl Trop Dis. 2024 Dec 2;18(12):e0012138. doi: 10.1371/journal.pntd.0012138 (PMC11637278; doi:10.1371/journal.pntd.0012138)
Supplement: Attachment — Submitted filename: Reviewers report.pdf [file pntd.0012138.s005.pdf]

Reviewer's report :

Title : Three years of insecticide resistance evolution and associated mechanisms in Aedes aegypti populations of Ouagadougou, Burkina Faso.

Effective date: 07 May 2024

Dear Editor

The present study concerns a three-year monitoring of Ae. aegypti susceptibility to insecticides and associated kdr mutations in two study sites in Ougadougou, Burkina Faso, a country where dengue epidemics regularly occur.

The results revealed an increasing level of resistance to permethrin and deltamethrin in the two study sites over the three years. Resistance was associated with the 1534C mutation, which was almost fixed throughout the three years in each site, and the 410L and 1016I mutations, which also increased significantly in frequency between 2016 and 2018. Interestingly, this study showed that Ae. aegypti populations in both study sites remained susceptible to bendiocarb, fenitrothion and malathion after these 3 years.

In general, the manuscript is written in acceptable English but requires proofreading.

Minor Comment

The submitted manuscript does not include line numbering, which makes it difficult to make precise reference to comments in the text. Authors should take this into account in future submissions.

In Figure S1 a and b of the 1200LG study site, the V/V genotypes are missing. The authors should correct this part.

On page 16, we suggest that authors write long-lasting insecticidal nets (LLINs) in full and not use an abbreviation the first time it is mentioned in the text.

Major Comment

In this study, the authors mentioned in the results and discussion that PBO activity decreased in correlation with an increase in resistance over the 3 years. However, according to Figure 1 c and d, pre-exposure to PBO showed an increase in deltamethrin mortality from ~80% to 98% (1.22-fold) in 2016; from ~10% to 80% (8-fold) in 2017 and from ~5% to 70% (14-fold) in 2018 in the 1200 LG locality. In Tabtenga, pre-exposure to PBO also increased deltamethrin mortality from ~50% to 98% (1.96-fold) in 2016; from ~20% to 90% (4.5-fold) in 2017 and from ~3% to 70% (23-fold) in 2018. The same trends were observed with permethrin and PBO in both sites.

Could the author provide a more detailed explanation for the decrease in oxidase activity mentioned in the manuscript over the three years?
