# Supplementary material for: Three years of insecticide resistance evolution and associated mechanisms in Aedes aegypti populations of Ouagadougou, Burkina Faso
Source: PLoS Negl Trop Dis. 2024 Dec 2;18(12):e0012138. doi: 10.1371/journal.pntd.0012138 (PMC11637278; doi:10.1371/journal.pntd.0012138)
Supplement: Attachment — Submitted filename: review2_.pdf [file pntd.0012138.s006.pdf]

PNTD-D-24-00505

review of Yameogo et al 2024:

Three years of insecticide resistance evolution and associated mechanisms in Aedes aegypti populations of Ouagadougou, Burkina Faso.

General comments:

In this paper, the authors evaluate the insecticide susceptibility of Ae. aegypti via metabolic and target site mechanisms over a 3-year surveillance period. For this they constituted 2 populations originated from 2 locations (Tabtenga and 1200LG). They performed bioassays to determine the susceptibility of the 2 populations to several insecticides. They also look for the presence ofseveral classic kdr mutation frequency. The results clearly showed a high resistance to the pyrethroid insecticides and the presence of kdr mutation that increased over the 3 years study. The results showed that insecticides from other family could be used as alternatives to the pyrethroids.

In my opinion, the introduction should be improved and need some more information. For example, the authors mention vector control used against Aedes in the world and in Africa but are there any specific vector control interventions in Burkina Faso (adulticiding and / or larviciding / when)? Same, is it the first time that insecticide resistance levels and their mechanisms are measured/detected in Burkina Faso?

This study was carefully planned and conducted and the results are clearly presented in 2 figures and 3 tables. The paper is well written, the background of dengue epidemics by both *Ae. aegypti* and *albopictus* in central Africa is well documented as well the insecticide resistance mechanisms in these specific populations. I recommend this paper with minor revisions for publication in PNTD.

Minor comments:

Introduction:

More information on vector control in Burkina Faso are needed

Are there any previous studies on IR in BF?

The paragraphs regarding the kdr mutation should be regrouped in the introduction.

The metabolic resistance mechanism should be more detailed.

“Many reports…lacking” this sentence should be rephrased, it is not clear

Methods:

It would be interesting to know to have an estimation of the number of larvae/pupae collected in the 2 localities to understand if the levels of resistance are coming from only few female mosquitoes or from more individuals. Is the number of individuals representatives of a large sampling area.

Why 2 different tests (WHO tube and CDC bottle) were used? The authors should explain this in the methods

Discussion:

The insecticide resistance levels were already high in 2016 indicating an intensive previous exposure to pyrethroids. The authors state that this could come from the treated Bednet distributions to target malaria vector. However Aedes aegypti is a day biting mosquito so the link between bednet presence and insecticide development in Aedes aegypti is not so obvious. Again vector control methods against Aedes mosquitoes but also against Anopheles and Culex should be more developed in the discussion. This would make the discussion clearer.
