# Supplementary material for: Three years of insecticide resistance evolution and associated mechanisms in Aedes aegypti populations of Ouagadougou, Burkina Faso
Source: PLoS Negl Trop Dis. 2024 Dec 2;18(12):e0012138. doi: 10.1371/journal.pntd.0012138 (PMC11637278; doi:10.1371/journal.pntd.0012138)
Supplement: Attachment — Submitted filename: Responses to Reviewers_140714.docx [file pntd.0012138.s007.docx]

**Responses to Reviewers comments**

In addition to reviewers comments, I also suggest to change [*] for [NS] to indicate an absence of significance in Fig1 in order to avoid misinterpretation of the data. Finally, as suggested by reviewers, I also believe that this MS deserves to be proofread by a native English speaker before being published.

- In Fig.1, we replace [*] with [NS].

The paper has been thoroughly proofread and corrected where necessary by a native English speaker.

Reviewer #1: The article “Three years of insecticide resistance evolution and associated mechanisms in Aedes aegypti populations of Ouagadougou, Burkina Faso”  gives an interesting insight on resistance evolving over a three year period in two sites within Ouagadougou. A large number of bioassays has been conducted and specimens genotyped for different pyrethroid resistance mutation. Results allow to appreciate the speed of resistance evolving under strong selective pressure. I would suggest the paper for publication and would recommend some minor corrections:

INTRODUCTION:
“The V1016I mutation confers resistance to pyrethroid insecticides only when associated with the F1534C mutation”: Please specify that this is known for Ae. aegypti; for other species few data are available on this.

We are grateful to the reviewer for the positive feedback about the manuscript.

We have added the required precision, the sentence is now “The V1016I mutation confers resistance to pyrethroid insecticides only when associated with the F1534C mutation in *Aedes aegypti* “

MATERIALS & METHODS :
As stated by you most of the dosages you use were revisited by WHO recently; I would suggest to mention this in discussion since you may underestimate levels of resistance for some of your populations.

- In the Discussion, we point out that most of the doses used in our study have recently been revised by the WHO, stating that ‘Most of the doses used in our bioassays where Anopheles doses, slightly higher than Aedes ones, providing a conservative assessment of *Aedes aegypti* resistance. Recently these doses have been revised by the WHO.[29].’

Why did you perform synergist assays using a different test protocol? This may be relevant also because you obtain very different results for pyrethroid bioassays at 1200Logements when using WHO-tube-test or CDC-bottle tests.

Our synergist assays were performed using the bottle bioassay protocol because in 2016, we didn't obtain the synergist-impregnated papers in time. We have added this information to the Methods. The reason of differences between CDC-bottles bioassay and WHO-tubes may be due to the fact that our bottle bioassays used specific doses for Aedes while our tube bioassays used Anopheles doses, which were higher than the doses for Aedes. As mentioned above specific, validated doses for Aedes were not available at the time of testing.

RESULTS

In the genotyping section and within the table referring to genotyping results please specify the number of specimens successfully genotyped. It is not clear whether you genotyped only specimens exposed to pyrethroids for pyrethroid resistance alleles or whether you genotyped all sampled specimens. This is relevant when interpreting results on the evolution of resistance alleles and the association of resistance alleles /genotypes/haplotypes with resistance phenotypes. It is obviously reasonable to include only genotyping results obtained for specimens exposed to pyrethroids when referring to the impact different resistance alleles /genotypes/haplotypes have on the possibility to survive pyrethroid exposure, but when referring to the evolution of resistance alleles /genotypes/haplotypes over the three year range it is definitely helpful to include as many specimens as possible ( and thus also those exposed to other insecticidal classes). “The frequency of the CIL/CIL triple mutant genotype increased from 2016 to 2018 (S1table) in 1200LG and Tabtenga, whereas the CVV/CVV genotype frequency decreased in both localities between 2016 and 2018 (S1table).” please add frequencies in the text to avoid to look them necessarily up in S1.

- The number of specimens is now specified in the Methods (‘A total of 286 *Aedes aegypti* samples were genotyped over three years: 94 samples in 2016, 98 samples in 2017 and 94 samples in 2018.’) and in S2Table.

- Pyrethroid-exposed specimens were genotyped for the years 2017 and 2018, but control mosquitoes (untested) were used in 2016 mosquitoes. This is now specified in the Methods.

- The frequencies of CIL/CIL and CVV/CVV genotypes showed in S1Table have been integrated into the text in the section ‘Evolution of F1534C, V1016I and V410L haplotypes, genotypes and their association with pyrethroid resistance.

DISCUSSION
Please rephrase the final sentence of the discussion section; it seems somehow contradictory since you already state before that LLIN probably have a high impact on selecting resistance.

- We rephrased the last sentence of the Discussion to state that ‘Recent studies have shown that *Ae. aegypti* exhibits exophilic but more likely endophagic behaviour [44] meaning that insecticide-treated bednets and other indoor-used insecticides may have limited contribution to the development of insecticide resistance.’

Reviewer #2: In general, the manuscript is written in acceptable English but requires proofreading.

The submitted manuscript does not include line numbering, which makes it difficult to make precise reference to comments in the text. Authors should take this into account in future submissions.

- The manuscript has been proofread and includes line numbering in its current version.

In Figure S1 a and b of the 1200LG study site, the V/V genotypes are missing. The authors should correct this part.

- We have corrected by including V/V genotypes in SFig1.a-b

On page 16, we suggest that authors write Long-Lasting Insecticidal Nets (LLINs) in full and not use an abbreviation the first time it is mentioned in the text.

- Long-Lasting Insecticidal Nets is written in full in the manuscript where first used.

In this study, the authors mentioned in the results and discussion that PBO activity decreased in correlation with an increase in resistance over the 3 years.
However, according to Figure 1 c and d, pre-exposure to PBO showed an increase in deltamethrin mortality from ~80% to 98% (1.22-fold) in 2016; from ~10% to 80% (8-fold) in 2017 and from ~5% to 70% (14-fold) in 2018 in the 1200 LG locality. In Tabtenga, pre-exposure to PBO also increased deltamethrin mortality from ~50% to 98% (1.96-fold) in 2016; from ~20% to 90% (4.5-fold) in 2017 and from ~3% to 70% (23-fold) in 2018. The same trends were observed with permethrin and PBO in both sites.

Could the author provide a more detailed explanation for the decrease in oxidase activity mentioned in the manuscript over the three years?

- We are grateful to the reviewer for pointing a misinterpretation of the PBO activity evolution over the three years. Taking into account the absolute mortality, we noticed that the mortality rate of PBO+deltamethrin decreased over years. But if we look into details the relative mortality of PBO compared to pyrethroid only, we observed an increase. That means that the synergistic effect of PBO increased and so the oxidase activity. We included the following sentence in the Discussion for more clarity: ‘The absolute mortality rate associated with PBO+pyrethroid decreased over the three year period, but the relative mortality attributable to PBO synergistic effect has increased. This indicates an increased synergistic effect of PBO, likely indicating heightened oxidase activity.’

Reviewer #3: In this paper, the authors evaluate the insecticide susceptibility of Ae. aegypti via metabolic and target site mechanisms over a 3-year surveillance period. For this they constituted 2 populations originated from 2 locations (Tabtenga and 1200LG). They performed bioassays to determine the susceptibility of the 2 populations to several insecticides. They also look for the presence of several classic kdr mutation frequency. The results clearly showed a high resistance to the pyrethroid insecticides and the presence of kdr mutation that increased over the 3 years study. The results showed that insecticides from other family could be used as alternatives to the pyrethroids.

In my opinion, the introduction should be improved and need some more information. For example, the authors mention vector control used against Aedes in the world and in Africa but are there any specific vector control interventions in Burkina Faso (adulticiding and / or larviciding / when)? Same, is it the first time that insecticide resistance levels and their mechanisms are measured/detected in Burkina Faso?

We developed a bit more the vector control activities implemented following dengue outbreak in Burkina Faso and we reported also the previous investigations on Aedes aegypti insecticide resistance in Burkina Faso.

Introduction: More information on vector control in Burkina Faso are needed. Are there any previous studies on IR in BF? The paragraphs regarding the kdr mutation should be regrouped in the introduction. The metabolic resistance mechanism should be more detailed.
“Many reports…lacking” this sentence should be rephrased, it is not clear

We have added information to the Introduction by including some details of dengue control vector control in Burkina Faso via the sentences ‘In Burkina Faso, dengue vector control has been implemented as an outbreak response since the 2016 outbreak and comprises of larval source management and insecticide spraying for vector control in dengue hotspots. Indoor and outdoor spraying was practiced in dengue patient houses and contiguous houses, and spatial spraying was used in public places and large larval sites, as a response to dengue outbreaks.’

We now provide coverage of previous *Aedes aegypti* resistance data in Burkina Faso as ‘In Burkina Faso, insecticide resistance of *Aedes aegypti* has been reported since 2016 with documented resistance to pyrethroids, moderate resistance to bendiocarb and susceptibility to malathion [8, 10, 19].’

We have regrouped the paragraphs on kdr mutations in the Introduction as suggested.

Data on metabolic resistance are sparse which we note by including the sentence ‘The mechanisms involved in pyrethroid resistance include the V1016I, 1534C and 410I kdr mutations [8; 19, 10], with which pyrethroid resistance is primarily associated and to a lesser extent metabolic resistance [8, 10, 11].’

Methods: It would be interesting to know to have an estimation of the number of larvae/pupae collected in the 2 localities to understand if the levels of resistance are coming from only few female mosquitoes or from more individuals. Is the number of individuals representatives of a large sampling area. Why 2 different tests (WHO tube and CDC bottle) were used? The authors should explain this in the methods

Larvae were collected in diversity of breeding containers in a minimum of 50 breeding containers and pooled in the laboratory per container type. The larvae were not counted but we can estimate the average number of larvae to 50 per breeding site. We think that the sample used for insecticide testing is representative of the population of the two localities.

We used different protocols - including bottle bioassays and WHO tube tests because in 2016, we didn't obtain the synergist-impregnated papers in time. We have added this information to the Methods.

Discussion: The insecticide resistance levels were already high in 2016 indicating an intensive previous exposure to pyrethroids. The authors state that this could come from the treated Bednet distributions to target malaria vector. However Aedes aegypti is a day biting mosquito so the link between bednet presence and insecticide development in Aedes aegypti is not so obvious. Again vector control methods against Aedes mosquitoes but also against Anopheles and Culex should be more developed in the discussion. This would make the discussion clearer.

We agree with the reviewer that the sources of selection pressure remain unclear. We cover sources of selection for resistance in Anopheles and Culex and the contrast with Aedes in the last paragraph of the Discussion, which we have reshaped to reflect our hypothesis of potential multiple sources of selection pressure at play in the domestic environment, whilst acknowledging the uncertainty and the requirement for further work.
